# Unraveling the Mechanism of Doping Borophene

**DOI:** 10.1002/open.202300121

**Published:** 2023-11-21

**Authors:** Kailash Pati Shiva Sankar Hembram, Jeongwon Park, Jae‐Kap Lee

**Affiliations:** ^1^ Center for Opto-Electronic Materials and Devices Korea Institute of Science and Technology Seoul 02792 Republic of Korea; ^2^ School of Electrical Engineering and Computer Science University of Ottawa, Ottawa Ontario K1N 6N5 Canada; ^3^ Department of Electrical & Biomedical Engineering University of Nevada Reno NV, 89557 USA

**Keywords:** borophene, doping, electronic properties, first-principles calculations, structure

## Abstract

We elucidate the doping mechanism of suitable elements into borophene with first‐principles density functional theory calculation. During doping with nitrogen (N), the *sp* ^*2*^ orbitals are responsible for arranging themselves to accommodate the electron of the N atom. Doping dramatically changes structure and electronic properties from corrugated and metallic borophene to flat and insulating h‐BN with 100 % N‐doping. We extend the mechanism of N‐doping in borophene to doping of non‐metallic and metallic ad‐atoms on borophene. Our findings will help to design boron‐based 2D materials.

## Graphical abstract

The nitrogen doping mechanism into borophene is investigated. The *sp* ^*2*^ orbitals of boron atoms in alternative triangular motifs are responsible for arranging themselves to accommodate the electron of N atom. While heavier atoms (Fe, Co, Ni) distort the local geometry of borophene, the lighter atoms (C, N, O) get rearranged.

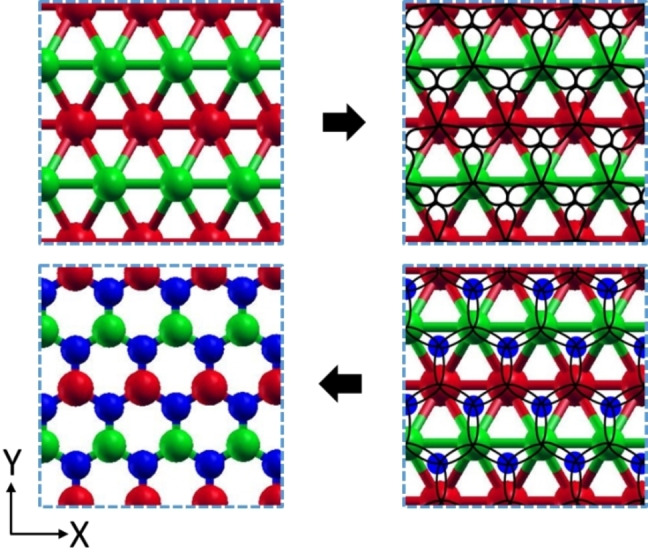

## Introduction

The synthesis of borophene,[^1^, ^2^] a monolayer of boron (B), opens an avenue to utilize B‐based two‐dimensional (2D) materials. Doping suitable elements into borophene can be considered one of the approaches to tune its bandgap. Dopants for borophene are not limited by their number of valence electrons, unlike the case of silicon. Nitrogen (N) is an ideal dopant, which with a favorable combination with B (B complex), can form 2D hexagonal boron nitride (*h*‐BN), being binary in nature. While borophene is corrugated and metallic in nature,[^1^, ^2^, ^3^, ^4^] *h*‐BN is planar and possesses a finite bandgap.[^5^, ^6^, ^7^] Thus, doping N (N‐doping) into borophene may be more complex than any other doping system. The immediate quest is to understand the fundamentals for doping borophene and know the feasibility of structure for suitable dopants, including N. In this study, we explore the doping behaviors of borophene with first‐principles density functional theory (DFT) simulation. Here we consider a triangular corrugated borophene (TCB) structure (Supporting Information Figure S1), simple, continuous in nature without vacancies.^[8]^ The choice of TCB (E_coh_=−6.65 eV/atom) is considered over another continuous structure, that is, triangular flat borophene (TFB), for being more stable (E_coh_=−6.44 eV/atom). However, it is reported that many borophenes appear as planar or corrugated[^8^, ^9^, ^10^, ^11^, ^12^, ^13^, ^14^, ^15^, ^16^, ^17^, ^18^, ^19^, ^20^, ^21^] in structure and clusters with regular or isolated vacancies and hollows, which are complicated in geometry.[^21^, ^22^, ^23^, ^24^, ^25^, ^26^, ^27^, ^28^, ^29^, ^30^, ^31^, ^32^, ^33^, ^34^, ^35^]

In the first part, we reveal the mechanism of N‐doping in borophene with the change in geometrical, electronic and vibrational properties. In the process, we show (i) the stability of the system (transforming the corrugated structure to planar), (ii) the variation of electronic property from metallic to insulating in nature, and (iii) the observation of phonon gap due to the mass difference between the constituent atoms, which let us reveal the mechanism of N‐doping into borophene. In the second part, we study the electronic properties of doped ad‐atoms (C, N, O, Fe, Co, Ni) in borophene. There are some investigations of non‐metallic elements doping on borophene, that is, O and H. The O‐doping in borophene nanoribbon changes the pristine metallic character to a semimetal or semiconductor character. The line edge shows high stability for finite‐size borophene nanoribbons compared to the zigzag edge.^[36]^ Studies have revealed that dual‐carbon doping in borophene is preferred for oxygen evolution reaction activity over single‐carbon doping in borophene.^[37]^ Hydrogenated borophenes give rise to borophanes. Interestingly, the low hydrogen coverage borophane shows a rhombic pattern, while the high hydrogen coverage one shows a rectangular pattern.^[38]^

The doping of *3d* elements is systematically carried out by various research groups.[^39^, ^40^, ^41^] However, their studies are not done on continuous triangular corrugate borophene motif, i. e., TCB, but on the borophene structure with central vacancies. Our investigations are in accordance with the literature protocol, and the results are compared in various instances.

## Results and Discussion

**Figure 1:**
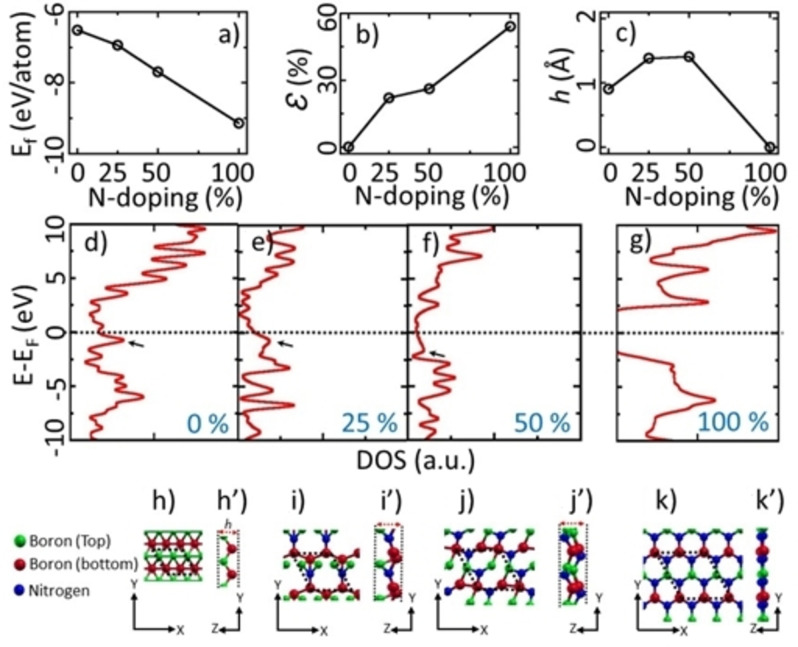
Evolution of the structure of N‐doped borophene. a) Formation energies (E_f_), b) lattice constant expansion (*Ɛ*), c) height (*h*) of corrugated structures, d–g) Electronic density of states of borophene, and N‐doped borophene, h–k) Top view, and h’–k’) side view of borophene and 25 %, 50 % and 100 % N‐doped borophene.

Figure 1a shows the formation energy (E_f_) of the chronological doping with N in borophene. The negative slope indicates that N‐doping is easier in borophene to form *h*‐BN. The lattice constant increases with the concentration of N, as shown in Figure 1b. For 25 % N‐doping, the pristine corrugated structure is distorted with a 22 % increase in lattice constant. N atom being heavier in mass and more electronegative (3.0) compared to that of B (2.0), tries to pull the electrons from adjacent B atoms.^[42]^ With the increase in 50 %‐ and 75 %‐N similar trend is observed. The lattice constant increases to 54 % with 100 % doping of N (*i. e*., *h*‐BN), leading to a dramatic structural change.

Comparing the structural parameters of borophene with *h*‐BN, we note the change in lattice constant from *a*_*1=*_1.61 Å, *a*_*2=*_2.86 Å to 2.50 Å (Table 1). Similarly, the B−B bonds (B1−B2=1.87 Å and B1−B1 (B2−B2)=1.61 Å) break to form B−N bonds with bond lengths of 1.44 Å. The intrinsic corrugation height (*h*) of 0.91 Å for borophene initially increases with doping, then decreases, finally becoming planar *h*‐BN (Figure 1k’) as shown in Table 1. Hence, with N‐doping in borophene, the structural anisotropy is lifted with the vanishing stripe structure, and planar geometry emerges with a homogeneous distribution of B and N atoms.

**Table 1:** Cohesive energy, E_coh_ and structural parameters of borophene, *h*‐BN.

Structure	E_coh_ [eV/atom]	Lattice constant [Å]		Bond length [Å]			height [Å]
		a1	a2	B1−B1	B2−B2	B1−B2	
TCB	−6.65	1.610	2.863	1.610	1.610	1.866	0.907
*h*‐BN	−9.14	2.501	2.501	1.445 (B−N)	–	–	0.000

Figures 1d‐g show the evolution of electronic structure through the total electronic density of states (DOS) from borophene to *h*‐BN with an increasing doping concentration of N. Pristine borophene is metallic while *h*‐BN is insulating. 25 % and 50 % N‐doped borophene keep metallic character, revealing finite DOS at the Fermi level. It is observed that the prominent peak of borophene (arrow in Figures 1d–f) in the valance band near the Fermi level diminishes with an increase in N‐doping and vanishes for *h*‐BN. The structural change while doping is illustrated in Figure 1h‐k. Due to the formation of *h*‐BN for 100 % N‐doping, dramatic structural change (from corrugated to flat), is evident.

**Figure 2:**
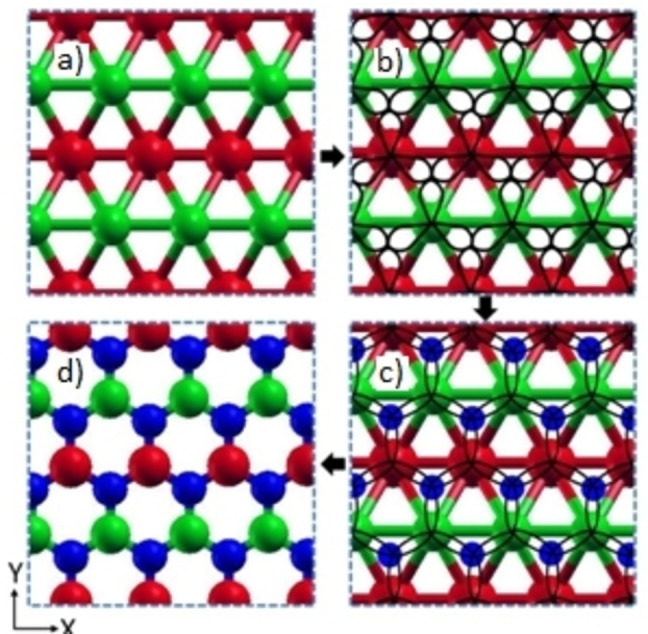
Mechanism of N‐doping in borophene and the structural transition from borophene to *h*‐BN. a) Borophene, b) Unoccupied orbital representation of borophene, c) N atom at the alternative triangular motif (distortion of orbitals with the formation of B−N bonding and breaking of B−B bonding), and d) *h*‐BN.

From the results shown in Figure 1, we draw a mechanism for N‐doping in borophene. B atom, with three valence electrons, possesses a *2s* ^*2*^, *2p* ^*1*^ electronic configuration. Each atom has six nearest neighbors in TCB, but in two adjacent corrugated sheets, with only three valence electrons. They form a three‐center bonding model in TFB, and we consider a similar model with slight differences in the TCB case. Additionally, a mixture of in‐plane and out‐of‐plane hybrid states exists due to buckling in TCB, which can be considered a symmetry‐reducing distortion that enhances binding. Hence, some energy states move below E_F_ as indicated by the small peak immediately below E_F_ (arrow in Figure 1d).^[8]^ Hence, some unoccupied *sp* ^*2*^ states partially share the electrons with the occupied states, directly making the corrugated structure. These unoccupied *sp* ^*2*^ states are prone to accept electrons (Figure 2b). When an N atom approaches borophene, it shares the electrons in the unoccupied *sp* ^*2*^ states. Thus, sharing an electron in orbitals makes new B−N bonds, breaking the B−B bonds (Figures 2c and 2d). With an increase in 100 % N‐doping (*i. e*., *h*‐BN), the bonding and antibonding orbitals open a gap at the Fermi level, as shown from the electronic DOS of the B_x_N_y_ complex (Figure 1g).

**Figure 3:**
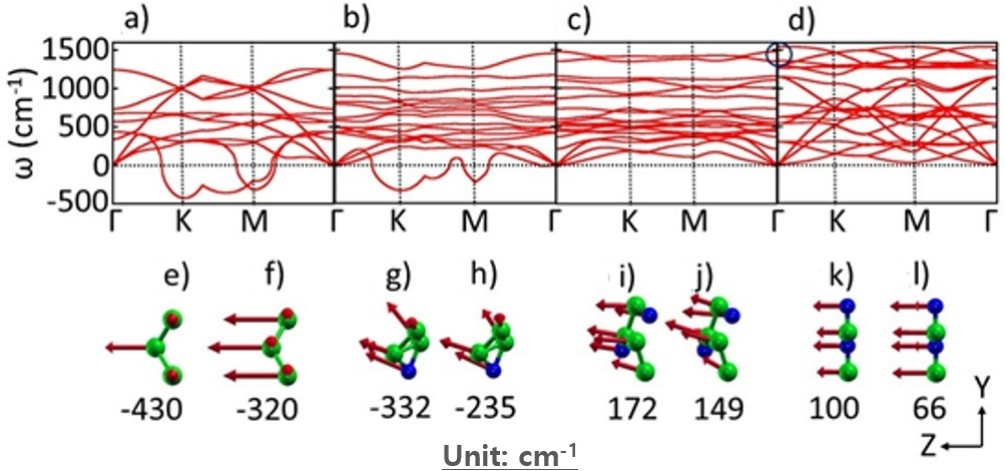
Phonon band structure of a) borophene, b‐d) 25 %, 50 % and 100 % N‐doped borophene, e, g, i, k) Flexural mode at K point of borophene and 25 %, 50 % and 100 % N‐doped borophene, and f, h, j, l) Flexural mode at M point of borophene and 25 %, 50 % and 100 % N‐doped borophene.

Their phonon band structures reveal the stability of the structures. Figure 3a‐d shows the evolution of the phonon band structure of N‐doping in borophene. While pristine borophene is unstable (due to negative phonon frequencies, Figure 3a), *h*‐BN reveals its stability without negative frequency in full phonon band structure (Figure 3d).^[5]^ We attribute borophene's negative flexural mode (acoustic modes with out‐of‐plane polarization, ZA) to its corrugated structure, supported by other experimental scanning tunneling microscope images and theoretical calculations.[^1^, ^2^, ^3^, ^4^] With 25 % N‐doping, the negative ZA mode shifts upward (−430 cm^−1^ to −332 cm^−1^ at K point and −320 cm^−1^ to −235 cm^−1^ at M point), and with 50 % doping, they are in the positive domain, showing full structural stability.

Figures 3e and 3 f show the flexural modes at the K‐point (−430 cm^−1^) and M‐point (−320 cm^−1^) in the Brillouin zone of borophene, respectively. While some flexural modes of B atoms are out‐of‐plane, some are directed perpendicular to the armchair direction, and this explains the appearance of the unique corrugated structure of borophene. For 25 % N‐doping, the flexural modes of B and N atoms have deviated from out‐of‐plane at both K‐point (−332 cm^−1^) and M‐point (−235 cm^−1^) (Figures 1g and 1 h). The eigenmodes of these anisotropic phonons make the structure unstable. With 50 % N‐doping, the flexural modes are fairly directed out‐of‐plane at K‐point (172 cm^−1^) and M‐point (149 cm^−1^) (although not perfectly), indicating relative structural stability. But for *h*‐BN, the flexural modes are perfectly directed out‐of‐plane, demonstrating complete 2D isotropic structural stability. No phonon gap is observed for pristine borophene (Figure 3a). But with N‐doping, the phonon gap is observed in a higher optical range of the entire phonon dispersion (Figures 3b‐d). Optic modes of *h*‐BN are split due to long‐range interactions (longitudinal optic (LO) ‐ transverse optic (TO) splitting) by 170 cm^−1^ (encircled at Γ‐point at the higher optical range for Figure 3d),^[5]^ which is unique to *h*‐BN but absent in borophene. The vanishing negative flexural mode and appearance of phonon gap in optical modes are the consequences of N‐doping, leading to the structural transformation, as shown in Figures 1h–k.

We extend the investigation of doping behavior for other non‐metallic (C and O) and metallic (Fe, Co, and Ni) elements in borophene. We have taken atomic species, as it is easier for experimentalists to consider doping through implantation (although expensive at present, but controlled). Various suitable positions were considered, such as top, bridge, and hollow sites on borophene (Supporting Information Figure S2). A suitable structure is obtained by comparing all ad‐atom doped borophene's binding energy (E_b_) and choosing the most stable optimized geometry among all configurations. The calculated binding energy and bond distance of the most suitable geometry is listed in Table 2 (details in Supporting Information Tables ST2, ST3 and ST4).

**Table 2:** Binding energies, E_b_ (eV/atom), and B/ad‐atom distance (Å) of the most stable optimized systems (2.77 % doping).

	C	N	O	Fe	Co	Ni
E_b_	−0.138	−0.178	−0.212	−0.218	−0.184	−0.169
*d*	1.640	1.329	1.430	1.945	1.945	2.053

**Figure 4:**
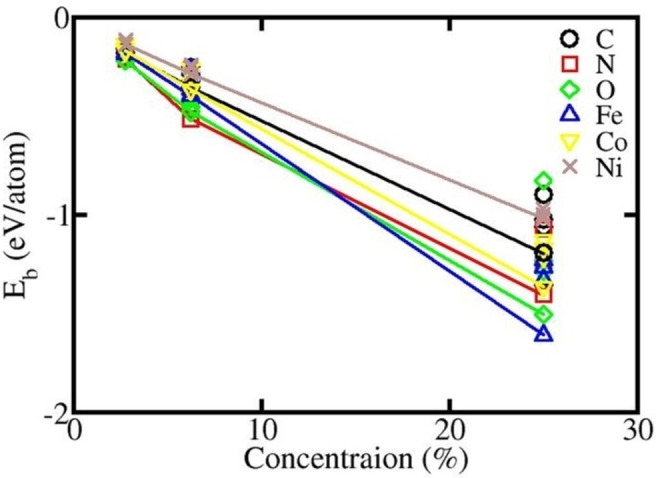
Doping of non‐metallic and metallic ad‐atoms in borophene.

N and O prefer the bridge site between two B atoms dragging them and making the structure more corrugated, while others prefer the hollow site and bonding with the four nearest B atoms. All ad‐atoms interact with surrounding B atoms of borophene so the local geometry rearranges. A similar trend is observed for higher‐concentration doping. Table 2 shows the shortest distances between the ad‐atom and the surrounding B atom for 2.77 % doping structures (details in Supporting Information Table ST4). Moreover, due to the large binding energies, all the transition metal atoms bind tightly with the substrate of the borophene sheet. This means the structures are very stable when the atoms are adsorbed on the surface of borophene.^[43]^ The negative slope of the binding energy curve indicates that doping is favorable at higher concentrations, as shown in Figure 4.

It is observed that 25 % Fe‐borophene and O‐borophene are more stable than N‐borophene compared to doping at 6.25 %. We attribute these, to the retention of optimized structures which are less distorted and more energetically favorable (from where the calculated energy results are taken), than other Fe‐borophene and O‐borophene geometries, which are highly distorted with less energetically favorable.

**Figure 5:**
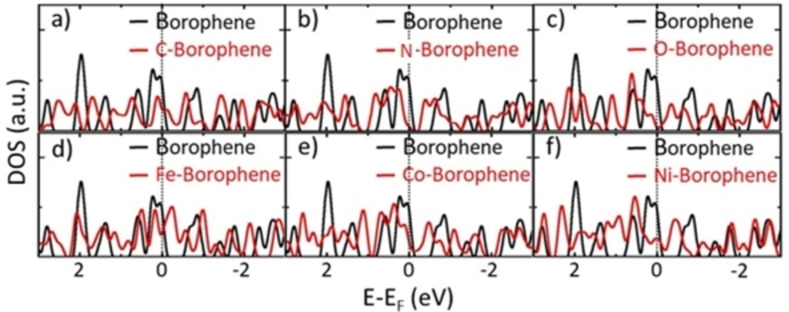
Electronic density of states of a) C, b) N, c) O, d) Fe, e) Co, and f) Ni doped borophene (2.77 % doping).

**Figure 6:**
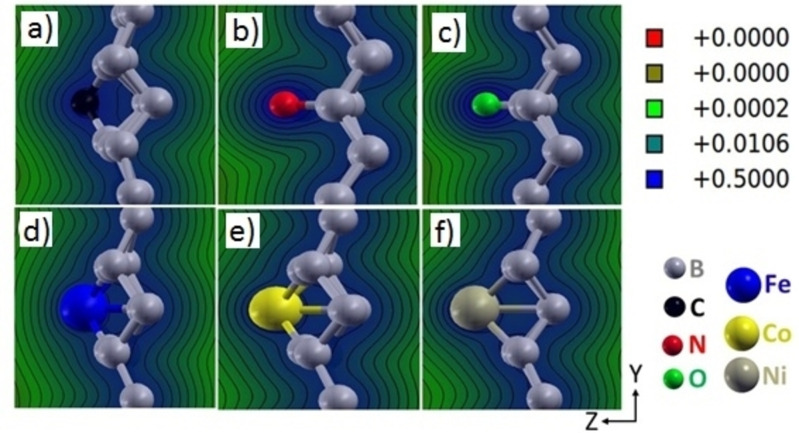
Isoelectronic contour plot of a) C, b) N, c) O, d) Fe, e) Co, and f) Ni doped borophene (2.77 % doping).

Figure 5 shows the electronic DOS of ad‐atoms on borophene. All the structures, along with borophene, show metallic character. The DOS decreases at the Fermi level for 2.77 % N‐ and O‐doped borophene, reducing their metallicity. Similar behaviors are observed from their 25 % doping (Supporting Information Figure S3). The iso‐electronic contour plot in a cross‐sectional view for the y‐z plane (Figure 6) elucidates the charge redistribution between ad‐atoms and borophene. The charge is accumulated more on C, N, and O than on borophene (Figures 6a–c) due to their higher electronegativity than B.^[42]^ The reverse trend is observed for Fe‐, Co‐ and Ni‐doped borophene (Figures 6d–f).

Based on the above results, we generalize the doping mechanism as follows. (i) The unoccupied *sp* ^*2*^ orbitals of the B atom at the alternative triangular site of borophene are active. (ii) Electrons from the foreign atoms approach the *sp* ^*2*^ orbitals. (iii) With the formation of new bonds, the structure rearranges and new electronic properties reveals.

## Conclusions

In summary, the unoccupied *sp* ^*2*^ orbitals of atoms of borophene are responsible for doping non‐metallic (N, C, O) and metallic elements (Fe, Co, Ni). With the rearrangement of fully occupied *sp* ^*2*^ orbitals, N‐doping in borophene stabilizes the system, resulting in dramatic changes in structure (corrugated borophene to planar *h*‐BN for 100 % N‐doping) and electronic property (metallic to insulating). Our findings will act as a guideline for designing other 2D materials.

## Computational Methods

Our calculations are based on first‐principles DFT as implemented in the Quantum Espresso (QE) simulation package.^[44]^ Generalized gradient approximation (GGA) was used for the exchange‐correlation energy of electrons.^[45]^ Ultra‐soft pseudopotentials were used to represent the interaction between ionic cores and electrons.^[46]^ Kohn‐Sham wave functions were represented with a plane‐wave basis with an energy cutoff of 50 Ry and a charge density with a cutoff of 300 Ry. Integration over an irreducible Brillouin zone was performed with a suitable mesh of *k* points.^[47]^ Occupation numbers were smeared using the Methfessel‐Paxton scheme with a broadening of 0.003 Ry.^[48]^ Dynamical matrices in the Brillouin zone were computed using a perturbative linear response approach.^[49]^

## Supporting Information

The supporting Information contains choice of a suitable mesh of grid points, schematic diagram of various borophene structures without vacancies, various initial geometrical configurations for suitable doping in borophene, and electronic DOS of ad‐atom doped (2×2 supercell) borophene.

## Conflict of interests

There are no conflicts to declare.

1

## Supporting information

As a service to our authors and readers, this journal provides supporting information supplied by the authors. Such materials are peer reviewed and may be re‐organized for online delivery, but are not copy‐edited or typeset. Technical support issues arising from supporting information (other than missing files) should be addressed to the authors.

Supporting Information

## Data Availability

The data that support the findings of this study are available from the corresponding author upon reasonable request.
