# Supplementary material for: Unraveling the Mechanism of Doping Borophene
Source: ChemistryOpen. 2023 Nov 21;13(3):e202300121. doi: 10.1002/open.202300121 (PMC10924041; doi:10.1002/open.202300121)
Supplement: Supplementary file 1 — Supporting Information [file OPEN-13-e202300121-s001.pdf]

ChemistryOpen

Supporting Information

Unraveling the Mechanism of Doping Borophene

Kailash Pati Shiva Sankar Hembram,* Jeongwon Park, and Jae-Kap Lee*

1. Choice of a suitable mesh of grid points.

For systematic N-doping with borophene:

Electronic structure calculation.	: 24×24×1
Phonon (perturbative linear response calculation).	: 4×4×1

For systematic doping of non-metallic and metallic elements.

2×2 supercell (25.00% doping).	: 12×12×1
4×4 supercell (6.25% doping).	: 8×8×1
6×6 supercell (2.77% doping).	: 4×4×1

2. Schematic diagram of various borophene structures without vacancies.

Borophene possesses various allotropes in 2D with different motifs. Without any vacancies, only three types of continuous structures are possible, such as triangular corrugated borophene (TCB), triangular flat borophene (TFB), and hexagonal flat borophene (HFB), which are in order according to their stability.[8]

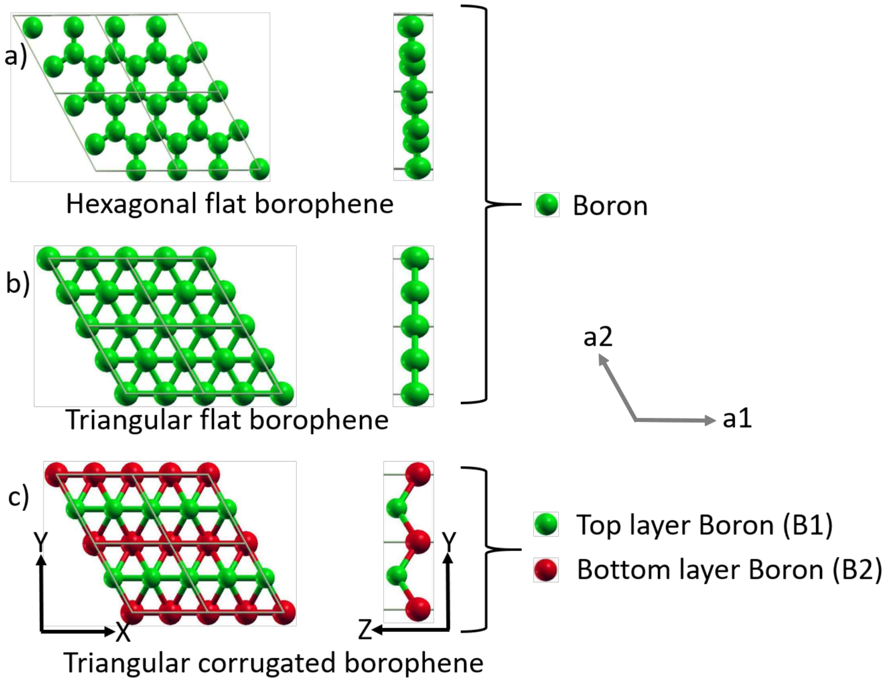

Figure S1. Schematic diagram (top and side view) of a) hexagonal flat borophene, b) Triangular flat borophene, and c) Triangular corrugated borophene (TCB).

Table ST1. Cohesive energies, E_{coh} (eV/atom), and structural parameters of various types of borophene.

	E_{coh} (eV/atom)	Lattice constant (Å)		Bond length (Å)			height (Å)
		**a1**	**a2**	B1-B1	B2-B2	B1-B2	
HFB	−5.65	2.91	2.91	1.678 (B-B)	-	-	-
TFB	−6.44	3.41	3.41	1.708	1.708	1.707	-
TCB	−6.65	3.25	3.25	1.610	1.610	1.866	0.907

($E_{coh} = E_{sheet} - E_{at}$, where E_{sheet} is the energy per atom of a sheet and E_{at} is the energy of an isolated boron/nitrogen atom).

Although we have written different terminologies, like E_f, E_{coh}, and E_b, for formation energy, cohesive energy, and binding energy, respectively, they are calculated using similar formulations. Lattice constant expansion ($\mathcal{E}$) = $(a_0\text{-}a)/a_0$, where ‘a_0’ is the pristine lattice constant and ‘a’ is the lattice constant after doping.

3. Various initial geometrical configurations for suitable doping in borophene

Ad-atoms can be doped at the “top site”, “atom-bridge site”, “hollow-bridge site” and “hollow site”. However, the relaxation process leads to the optimized site owing to the minimum energy.

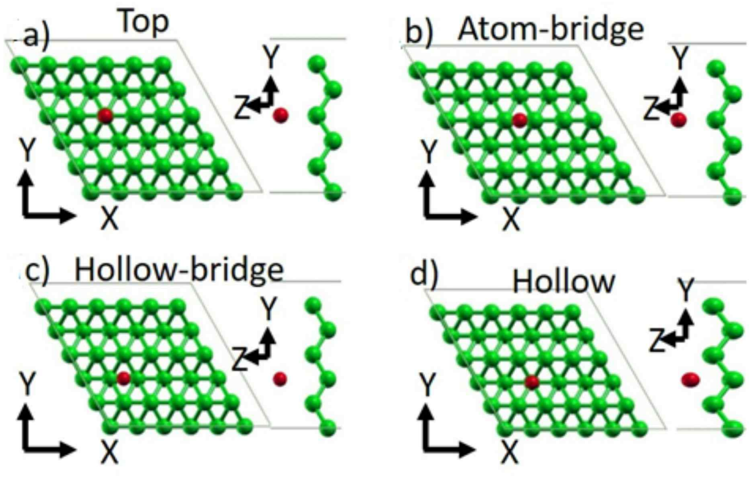

Figure S2. Various initial geometrical configurations for suitable doping for (6×6 supercell) borophene.

Table ST2. Binding energy, E_b (eV/atom), and boron/ad-atom distance (Å) of all optimized systems for 6×6 supercell with 2.77 % doping.

	C	N	O	Fe	Co	Ni
E_b(t)	−0.159	−0.212	−0.218	−0.157	−0.135	−0.119
E_b(at-br)	−0.159	−0.208	−0.218	−0.175	−0.160	−0.116
E_b(h-br)	−0.160	−0.202	−0.214	−0.184	−0.169	−0.137
E_b(h)	−0.178	−0.178	−0.216	−0.177	−0.163	−0.138
d(t)	1.535	1.329	1.430	1.913	1.937	1.944
d(at-br)	1.537	1.429	1.431	2.030	2.003	1.922
d(h-br)	1.506	1.423	1.414	1.945	1.945	1.957
d(h)	1.640	1.657	1.416	2.027	2.012	2.053

t=top, at-br =atom-bridge, h-br = hollow-bridge, h=hollow.

Table ST3. Binding energy, E_b (eV/atom), and boron/ad-atom distance (Å) of all optimized systems for 4×4 supercell with 6.25% doping.

	C	N	O	Fe	Co	Ni
E_b(t)	−0.256	−0.335	−0.411	−0.300	−0.286	−0.252
E_b(at-br)	−0.314	−0.513	−0.452	−0.265	−0.260	−0.241
E_b(h-br)	−0.354	−0.464	−0.479	−0.397	−0.364	−0.254
E_b(h)	−0.346	−0.364	−0.281	−0.362	−0.335	−0.286
d(t)	1.486	1.350	1.272	1.945	1.932	1.936
d(at-br)	1.553	1.316	1.432	1.929	1.906	1.920
d(h-br)	1.529	1.412	1.430	1.978	1.933	1.908
d(h)	1.628	1.680	1.817	2.029	2.019	2.052

t=top, at-br =atom-bridge, h-br = hollow-bridge, h = hollow.

Table ST4. Binding energy, E_b (eV/atom), and boron/ad-atom distance (Å) of all optimized systems for 2×2 supercell with 25% doping.

	C	N	O	Fe	Co	Ni
E_b(t)	−0.899	−1.071	−1.285	−1.263	−1.167	−0.996
E_b(at-br)	−1.024	−1.404	−1.505	−1.229	−1.128	−0.968
E_b(h-br)	−1.063	−1.258	−1.234	−1.608	−1.363	−1.017
E_b(h)	−1.193	−1.067	−0.828	−1.330	−1.212	−1.018
d(t)	1.572	1.383	1.278	1.969	1.936	1.958
d(at-br)	1.535	1.516	1.421	2.016	1.981	1.999
d(h-br)	1.518	1.426	1.405	1.980	1.946	1.956
d(h)	1.632	1.662	1.989	2.075	2.084	2.097

t = top, at-br = atom-bridge, h-br = hollow-bridge, h = hollow.

4. Electronic DOS of ad-atom doped (2×2 supercell) borophene.

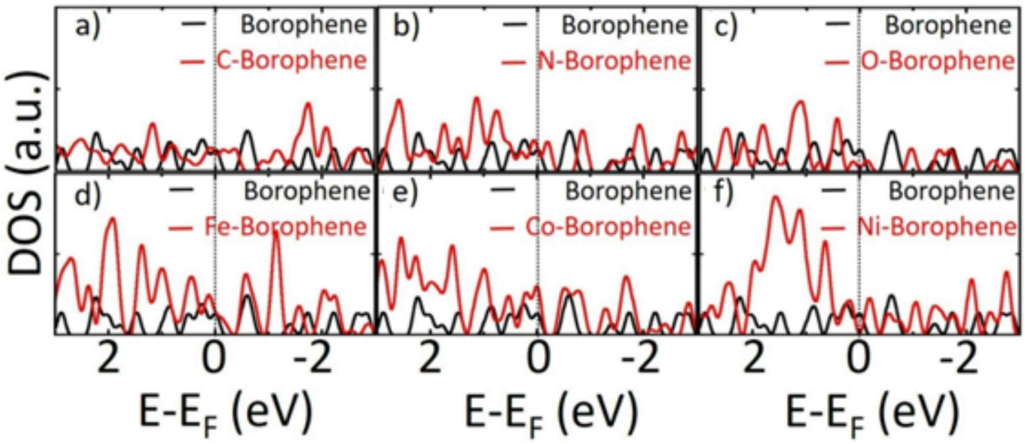

Figure S3. Electronic DOS of a) C, b) N, c) O, d) Fe, e) Co, and f) Ni doped (2x2 supercell) borophene.
